# Partial Monosomy 21 Mirrors Gene Expression of Trisomy 21 in a Patient-Derived Neuroepithelial Stem Cell Model

**DOI:** 10.3389/fgene.2021.803683

**Published:** 2022-02-04

**Authors:** Jakob Schuy, Jesper Eisfeldt, Maria Pettersson, Niloofar Shahrokhshahi, Mohsen Moslem, Daniel Nilsson, Niklas Dahl, Mansoureh Shahsavani, Anna Falk, Anna Lindstrand

**Affiliations:** ^1^ Department of Molecular Medicine and Surgery and Center for Molecular Medicine, Karolinska Institutet, Stockholm, Sweden; ^2^ Department of Clinical Genetics, Karolinska University Hospital, Stockholm, Sweden; ^3^ Science for Life Laboratory, Karolinska Institutet Science Park, Solna, Sweden; ^4^ Department of Neuroscience, Karolinska Institutet, Stockholm, Sweden; ^5^ Department of Immunology, Genetics, and Pathology, Uppsala University, Uppsala, Sweden; ^6^ Lund Stem Cell Center, Lund University, Lund, Sweden

**Keywords:** ring chromosome 21, trisomy 21, RNA-Seq, induced pluripotent stem cells, neuroepithelial stem cells, microarray, genomic deletion, chromosomal abnormalities

## Abstract

Induced pluripotent stem cells (iPSCs) from patients are an attractive disease model to study tissues with poor accessibility such as the brain. Using this approach, we and others have shown that trisomy 21 results in genome-wide transcriptional dysregulations. The effects of loss of genes on chromosome 21 is much less characterized. Here, we use patient-derived neural cells from an individual with neurodevelopmental delay and a ring chromosome 21 with two deletions spanning 3.8 Mb at the terminal end of 21q22.3, containing 60 protein-coding genes. To investigate the molecular perturbations of the partial monosomy on neural cells, we established patient-derived iPSCs from fibroblasts retaining the ring chromosome 21, and we then induced iPSCs into neuroepithelial stem cells. RNA-Seq analysis of NESCs with the ring chromosome revealed downregulation of 18 genes within the deleted region together with global transcriptomic dysregulations when compared to euploid NESCs. Since the deletions on chromosome 21 represent a genetic “contrary” to trisomy of the corresponding region, we further compared the dysregulated transcriptomic profile in with that of two NESC lines with trisomy 21. The analysis revealed opposed expression changes for 23 genes on chromosome 21 as well as 149 non-chromosome 21 genes. Taken together, our results bring insights into the effects on the global and chromosome 21 specific gene expression from a partial monosomy of chromosome 21qter during early neuronal differentiation.

## Introduction

Chromosome 21 is the smallest human chromosome including only 237 protein-coding genes (GRCh38/hg38). Although trisomy 21 (i.e. Down Syndrome; MIM #190685) is the most commonly seen chromosomal abnormality, occurring in 0.152% of live births (Moorthie et al., 2018), monosomy of chromosome 21 is not compatible with life (Schinzel, 1976; Burgess et al., 2014). In rare cases however, partial deletions of chromosome 21 have been reported (Lindstrand et al., 2010), and similar to patients with trisomy 21 (Wu et al., 2014), developmental delay, delayed motor function, and developmental heart defects are common clinical symptoms in such individuals. The clinical presentation associated with a partial monosomy will depend on the size and location of the deletion and the specific genes involved. When deletions are located close to or within the subtelomeric regions, the chromosomes may become unstable promoting rescue through chromatin fusions leading to chromosomal circularization and the formation of a ring chromosome.

Ring chromosomes are reported to spontaneously occur at a rate of 1:50,000 in newborn children (Jacobs et al., 1975) and frequently include additional structural genomic variants such as telomeric deletions, inversions or duplications (Nikitina et al., 2021). Though there is a wide phenotypic spectrum, ranging from completely healthy to severe pediatric diseases, the hallmark features of a proband carrying ring chromosomes, sometimes referred to as ring-syndrome, include a variable degree of growth failure, intellectual disability and developmental delay (Rossi et al., 2008; Yip, 2015; Nikitina et al., 2021). Furthermore, the phenotypic consequences of a specific ring chromosome may vary due to the unstable nature of the rearranged chromosome leading to additional genomic aberrations (Kosztolányi, 1987). The exact clinical presentation of the carriers will therefore depend on the combination of loss or gain of genes and the dynamics of the ring chromosome structure.

RNA-Seq is an excellent method to study genome-wide expression changes and to bring insights into complex dysregulations caused by structural variations (Cummings et al., 2017). However, many genes are not expressed in every tissue, is it therefore important to investigate expression in disease-relevant cells. This is particularly problematic for genes expressed in neural tissue since access to post-mortem brain biopsies is limited. Moreover, easier accessible tissues such as blood or fibroblasts have transcriptomic profiles that cannot be translated to that of neural cells (Aicher et al., 2020). To overcome this obstacle, somatic cells may be reprogrammed into induced pluripotent stem cells (iPSCs) to regain a pluripotent state followed by differentiation into neuronal cell types (D’Antonio et al., 2018). In any RNA-Seq experiments, the data will only contain transcripts from expressed genes. In consequence, relevant expression changes will be missed if an unsuitable cell type is used (Aicher et al., 2020).

In the present study, we used iPSC-derived neuroepithelial stem cells (NESCs) to model early neural development in a clinical case with neurodevelopmental delay and a partial monosomy of chromosome 21. We analyzed transcriptome profile in NESCs with ring chromosome 21 together with that in corresponding euploid cells and cells with a full trisomy 21. We present here the genome-wide effects caused by monosomy for 60 genes on chromosome 21qter.

## Material and Methods

### Ethical Statement

All studies were approved by the local ethical boards in Stockholm, Sweden (Genomic studies, Dnr 2012/2106-31/4 and cellular studies, Dnr 2016/430-31) and written informed consent was obtained.

### Human Subjects and Study Design

Patient RD_P26 was born as the second child to healthy non-consanguineous parents of Swedish origin. She was born at term, the pregnancy was uneventful, but an extra ultrasound was executed due to suspicion of poor growth. Birth weight was 2780 g (−2 SD) and birth length 48 cm (−1 SD), head circumference 32 cm. The girl was first referred for genetic investigation at age 2 years and 7 months due to hemifacial microsomia, feeding problems, speech delay, postnatal growth delay (−2 SD for both length and weight) and a preauricular appendage on the right ear. Clinical follow up at age nine revealed a normal cognitive level, persistent impressive and expressive speech delay, severe feeding difficulties that required PEG until age 6. Persistent postnatal growth delay (body length 125 cm, body weight 20 kg). Facial features showed a triangular face with mild hemifacial microsomia. Both vision and hearing were normal. Ultrasound of the heart revealed a structurally normal heart with a minor aorta insufficiency.

Two previously reported NESC lines (DS1 and DS2) (Sobol et al., 2019), both with a full trisomy 21 as well as the euploid NESC lines CTRL 7 (Kele et al., 2016; Lam et al., 2019), CTRL9 (Uhlin et al., 2017; Lundin et al., 2018) and CTRL11 generated from healthy donors (one female, two males), were included in our study (Supplementary Table S1).

### Array Comparative Genomic Hybridization Analysis

A 4 × 180K custom oligonucleotide microarray was used for array comparative genomic hybridization (aCGH) performed on peripheral blood (AMADID:03103, Oxford Gene Technology, Begbroke, Oxfordshire, United Kingdom) as previously described (Lindstrand et al., 2019). The probes were targeted genome-wide with a median probe space of 18 kb excluding telomeric and centromeric regions. Controls consisted of sex-matched healthy donors. Findings were grouped by ACMG (Riggs et al., 2020) and compared to DECIPHER (Firth et al., 2009) and the Online Mendelian Inheritance in Man database (Online Mendelian Inheritance in Man, OMIM, 2020).

### Fluorescence *in situ* Hybridization

Fluorescence *in situ* hybridization (FISH) was performed following a standard protocol on cultures derived from peripheral blood (Lindstrand et al., 2010). Four BAC probes targeting 21q22.2 and 21q22.3 were used for labelling (coordinates for GRCh38/hg38): RP11-446L19-SO (21: 42,816,935-43,130,340) and RP11-619i15-SO (21:42,221,275-42,410,371), RP11-53E17-SG (21: 43,477,579-43,653,469) and RP11-114H1-SO (21:40,817,025-40,979,068).

### Karyotyping

NESCs, iPSCs and cells from peripheral blood were cultured for karyotyping according to standardized protocol. The passage number for cells did not exceed 30. The chromosome analysis of the metaphase slides was performed after G-banding demonstrating a resolution of 550 bands per haploid genome. Results were based on a minimum of 25 mitosis per patient.

### Immunocytochemistry

The cells were fixed and stained according to protocol (Kele et al., 2016). Briefly, fixation was performed in 4% PFA for 10 min, blocked in 10% bovine serum/0.2% Triton/PBS for 1 h before the primary antibody was applied diluted 1:200 in blocking buffer at 4°C overnight. Primary antibodies: Oct-4A-rabbit (Cell Signaling technology, #2840), Nanog-rabbit (Cell Signaling technology, #4903), SOX2-rabbit (Sigma-Aldrich, #AB5603), E-Cadherin-mouse (BD BioSciences, #610181), PLZF-rabbit (Thermofisher Scientific, #PA5-29213) and Nestin-mouse (Merck, MAB5326). All secondary antibodies were diluted in PBS in 1:500 using blocking buffer. Secondary antibodies: Goat anti-mouse-Cyanine3 (Thermofisher Scientific, #M30010), Donkey anti-rabbit-Alexa fluor 488 (Thermofisher Scientific, #A-21206). Nuclei were stained with DAPI (Life Technologies, #D1306), diluted 1:5,000 in PBS for 30 min. Image acquisition was performed on a Axioskop 2 (Zeiss) and software Axiovision version 4.8.

### Generation of iPSC and NESC Lines

Skin biopsies were taken (all samples included in Supplementary Table S1) and fibroblast cultures were established following an enzymatic digestion protocol. Fibroblasts were reprogrammed to iPSCs with a non-integrating method described previously (Shahsavani et al., 2018). In brief, 0.1 × 10^6^ fibroblasts were reprogrammed by the overexpression of *OCT4*, *SOX2*, *C-MYC* and *KLF4* using *Sendai* viruses and cultured in Essential 8™ medium (Gibco). After 7 days, transduced cells were replated on Laminin-521 and emerging colonies were manually picked after 13 more days. Each colony was cultured onto a Lamin-521 coated plate and passage every 3–5 days by TrypLE-Select. Derived iPSC lines were checked for pluripotency markers OCT4 and NANOG.

The iPSC colonies were neurally induced to capture neuroepithelial stem cells (NESCs) (Falk et al., 2012). Briefly, neural induction was achieved by culturing the iPSCs in 0.5 ng/ml hNoggin and 3.3 µM CHIR99021 until day 10. The medium was supplemented with 10 µM SB431542 until day 4. The cells were replated at 40,000 cells/cm^2^ onto 0.1 mg poly-l-ornithine 1 μg/ml laminin L2020–coated plates in medium containing Dulbecco modified eagle medium/F-12 GlutaMAX, 1% N2, 0.1% B27, 10 ng/ml FGF2, 10 ng/ml EGF, and 1% Pen/Strep. NESCs were grown in a rosette-like morphology and stained positive for the neural stem cell markers SOX2 and NESTIN. Generated NESC lines were then cultured for several passages (>6) to ensure a homogenous culture without undesired cell types before cells were collected with TrypL-Express and harvested for DNA (DNA extraction: DNeasy Blood and Tissue Kit, QIAGEN, protocol 2016) or RNA (RNA extraction: Qiagen AllPrep DNA/RNA mini kit). All iPSC and NESC lines were created at the iPS Core facility at Karolinska Institutet Stockholm, Sweden.

All six NESC lines in the study such as RD_P26, with a ring chromosome 21, DS1 and DS2 lines with a full trisomy 21 (Sobol et al., 2019), and the euploid “control” NESC lines CTRL 7, 9 and 11 were cultured under similar conditions.

### RNA Analysis and Gene Expression

RNA-Seq was performed in technical triplicates on total mRNA from each of the six NESC lines at the National Genomics Infrastructure (NGI) in Stockholm using the Illumina TrueSeq Stranded mRNA kit; the resulting libraries were sequenced on the Nova-Seq 6000 platform, resulting in roughly 25M 2X150 bp reads per donor. Read sequences were used for transcriptome assembly. Derived data was stored in the FASTQ format in the UPPMAX compute infrastructure in Uppsala. For expression analysis, the FASTQ files were used as input for the bioinformatic tool Salmon V1.1.0 (Patro et al., 2017). It reads the FASTQ file, then estimates transcript-level abundance and returns a salmon file with information for each transcript found. Gene expression changes were calculated from the generated salmon files with an in-house R pipeline (github.com/jakschuy/RNASeq), based on the DeSeq2 R Script (Love et al., 2014). The required tx2gene list was derived from Gencode GRCh38.p13/hg38, release 36 (Frankish et al., 2019). All calculations in R were performed with R V4.0.3 in R Studios V1.2.5033. Gene annotations are extracted from BioMart - Ensembl for gene annotation (Kinsella et al., 2011).

Genes were defined as differentially expressed when fold-change exceeded ±25% when compared to the expression in euploid lines. The statistical approach in RNA-Seq analysis is inherited from the DeSeq2 package V1.26.0 (Love et al., 2014). In brief, the calculation for test significance is performed with the Wald test followed by a *p*-value correction with the Benjamini-Hochberg adjustment. The therewith derived adjusted *p*-value is filtered by a default cut-off of 0.1, representing the commonly used and accepted false discovery rate (FDR) of 10%. However, due to the small number of biological replicates in our study we narrowed the cut-off to 5%, filtering the adjusted *p*-value at 0.05. The result was further filtered against a base mean value below 5.

Permutation tests were performed on the output of DeSeq2 of line RD_P26 with n = 1,000. Subsequently, each permutation case data was processed equally to RD_P26-derived data regarding filtering and thresholds.

To analyze allele specific expression, the FASTQ file was processed by GATKs best practices for RNA-Seq. The data from the VCF file were filtered for high quality findings by excluding indels and entries with read counts representing coverage below 50. Applied R packages: tidyverse V1.3.0, cowplot 1.0.0, dplyr V1.0.5, purrr V0.3.3, ggrepel 0.8.1 and ggplot2 V3.3.3 (R script: github.com/jakschuy/RNASeq).

## Results

### Establishing a NESC Model From Patient-Derived iPSCs

**FIGURE 1:**
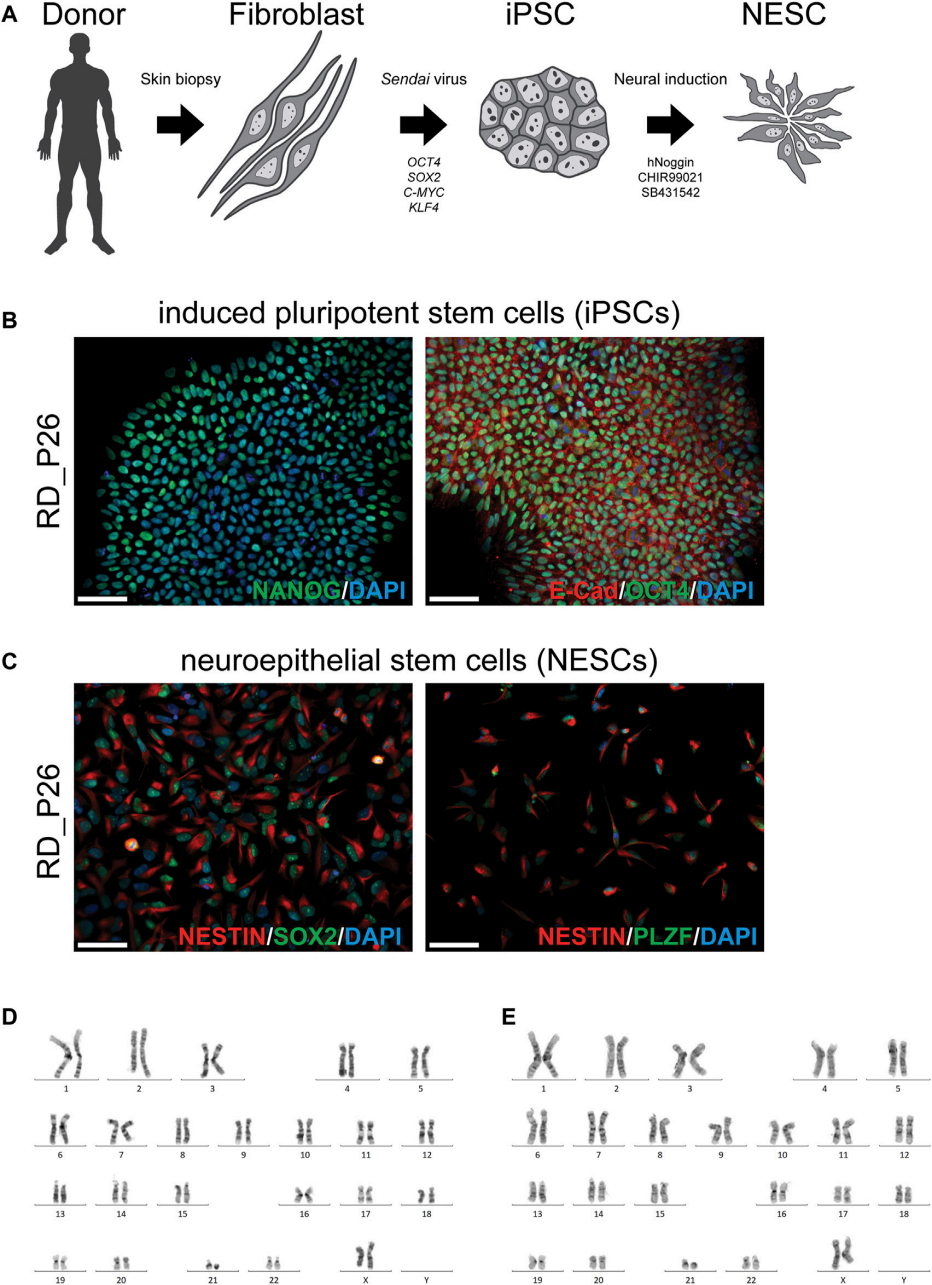
Generation of a patient specific cellular model and characterization of iPSC and NESC lines of RD_P26 **(A)** Fibroblasts are extracted from the donor and reprogrammed via overexpression of the transcription factor *OCT4*, *SOX2*, *C-MYC* and *KLF4* delivered *Sendai* virus vectors. After 28 days, iPSCs were neurally induced by supplementing the growth medium with hNoggin, CHIR99021 and SB431542. After 10 days post-induction rosette-like structures are observed **(B)** Patient iPSCs (RD_P26) used in this study expressing pluripotency markers E-Cadherin, OCT4 and NANOG **(C)** The iPSCs are neurally induced and give rise to neuroepithelial stem cells (NESCs) expressing neural specific markers NESTIN, SOX2 and PLZF. Nuclei are stained with DAPI. Scale bar 50 µm **(D, E)** The patient cell lines were karyotyped showing an abnormal chromosome 21 in iPSCs **(D)** and NESCs **(E)**.

To establish an *in vitro* cellular model for our continued studies, we obtained a skin biopsy from the patient (RD_P26). A fibroblast cell culture was established and subjected to reprogramming into iPSC using a non-integrating method (Figure 1A). The iPSCs stained positive for the pluripotency markers NANOG, OCT4 and E-CAD (Figure 1B) and subsequently induced to NESCs as described (Falk et al., 2012). The NESCs showed a rosette-like morphology and stained positive for the neural stem cell markers NESTIN, SOX2, and PLZF (Figure 1C). Proliferation, expansion, and growth pattern of NESCs from RD_P26 were compared to that of euploid NESCs and of the two independent NESC lines with trisomy 21 (Kele et al., 2016; Uhlin et al., 2017; Lundin et al., 2018; Lam et al., 2019; Sobol et al., 2019) showing similar viability (data not shown). Karyotypes of patient-derived iPSC and NESC lines showed a retained ring chromosome 21 (Figure 1D,E). The characterization of sample CTRL11 of iPSCs and NESCs is provided in Supplementary Figure S1A,B, respectively.

### Molecular Cytogenic Studies

**FIGURE 2:**
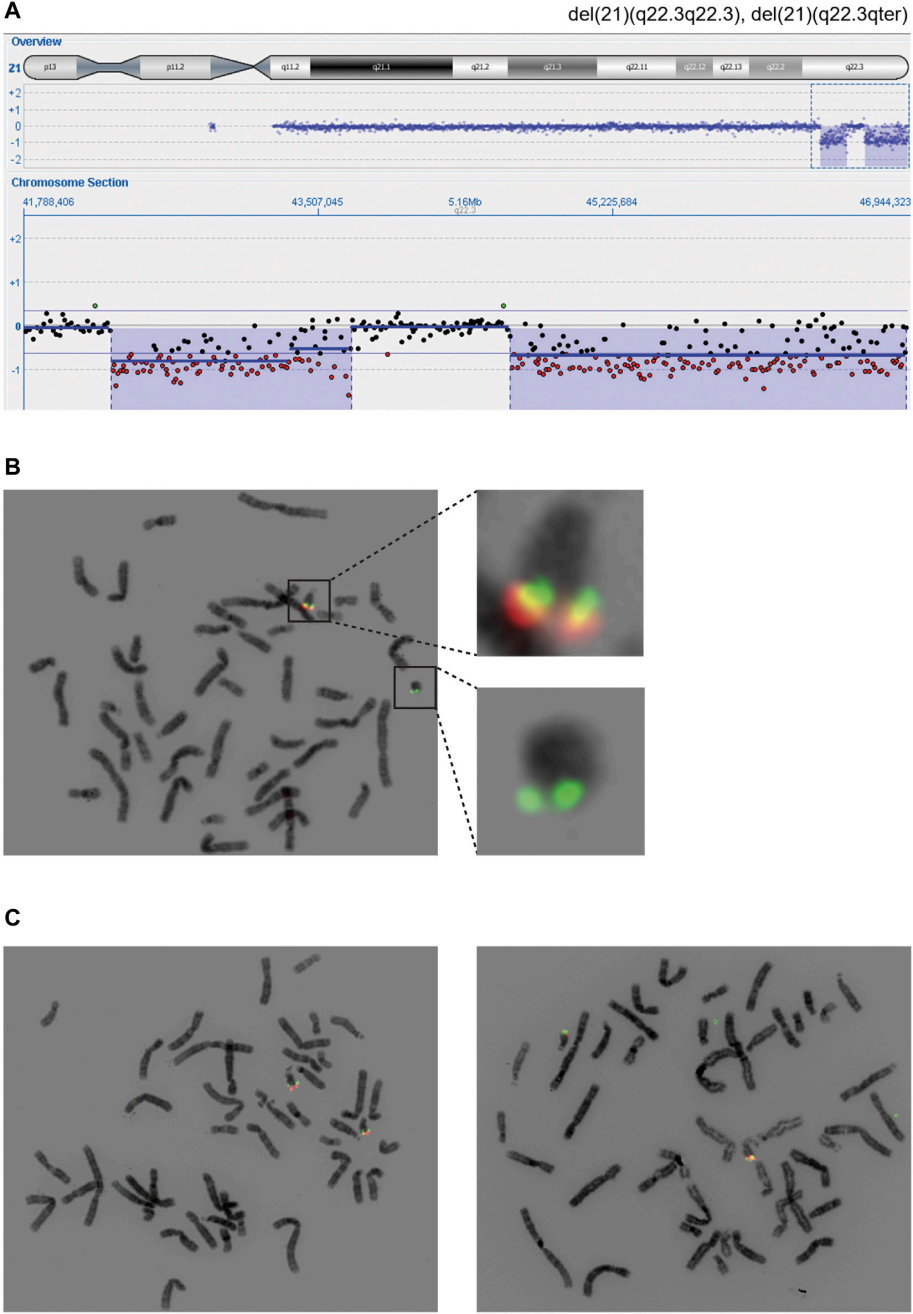
Cytogenetic findings revealing distal deletions on chromosome 21q **(A)** The 21qter deletions (blue) were found using array comparative genomic hybridization and visible due to the hemizygous detection level (red) **(B)** Fluorescent *in-situ* hybridization using probes within deletion one and 2 (red) and outside of deleted areas (green). Regions of interest are shown in higher magnification **(C)** Two probes (red, green) were used to confirm the homozygous state of distal regions on chromosome 21q in the maternal (left) and paternal (right) cell lines coming from peripheral blood. The probes used are identical with the ones in panel B and hybridize with a region in 21q22.3 (red) and 21q:22.2 (green).

Array comparative genomic hybridization (aCGH) performed on RD_P26 using DNA from blood demonstrated two terminal deletions on chromosome 21q22.3 (21:41,994,799-43,447,106; 21:44,361,567-46,944,323) (Figure 2A). Fluorescence *in situ* hybridization (FISH) analysis on metaphase chromosomes from blood using probes located adjacent to and within the respective locations confirmed the presence of both deletions and the finding from the karyotyping showing the presence of a ring chromosome 21 (Figure 2B). FISH of parents showed normal diagrams (Figure 2C).

### RNA-Seq Data Shows Downregulation for Genes Within Deleted Regions

RNA-Seq data was performed on bulk RNA from proliferating NESCs from RD_P26 with a ring chromosome 21 and three euploid control lines. The gene expression data was computed with DeSeq2. Genes with expression deviating more than 25% in RD_P26 when compared to expression in euploid NESCs were grouped as differentially expressed genes (DEGs).

**FIGURE 3:**
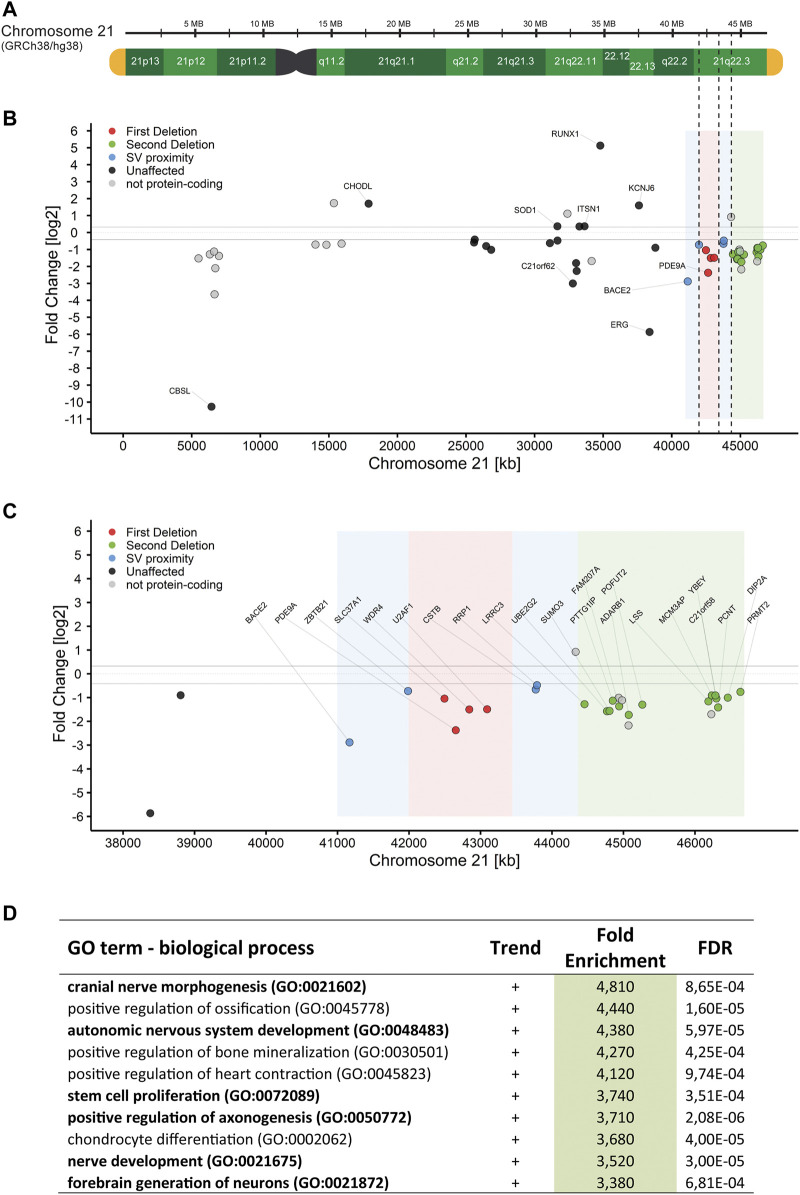
RNA-Seq of NESCs in RD_P26 reveals downregulated genes on chr21q **(A)** Graphical representation of chromosome 21 based on the human reference genome GRCh38/hg38, marked for the two deletions on chr21q (dashed) **(B)** Gene expression in RD_P26 of protein-coding genes on chromosome 21. Genes are colored according to their location in deletion 1 (red), deletion 2 (green) as well as the 1 Mb vicinity close to the SVs. Non-protein-coding genes are included (pale dots). The five highest and five lowest expressed genes are labelled **(C)** Gene expression in RD_P26, zoomed in on the distal region of chromosome 21. Color coding as in panel B. All protein-coding genes located in the deletions and close proximity are labelled **(D)** Top ten findings of the pathway analysis for differential expressed genes in RD_P26. Pathways related to brain tissue are marked in bold. Complete list can be found in Supplementary Table S3.

After quality filtering, the RNA-Seq data contained 14,246 protein-coding genes, 71% of all 20,093 protein-coding genes in GRCh38/hg38. Of those, 1951 (13.7%) genes were differently expressed (Supplementary Figure S2A, raw data in Supplementary Table S2). Notably, genes belonging to the *HOX* gene clusters A and B on chromosomes 7 and 17, respectively were upregulated between 26 and 2204-fold (median = 199-fold) in RD_P26. On chromosome 21, the RNA-Seq data detected 141 genes of the 237 protein-coding genes and 28% (40/141) were differently expressed when compared to the transcriptomes of euploid NESCs (Figures 3A,B). Among the 31 expressed genes in NESCs located in the two deleted regions of the ring chromosome, 18 were downregulated in RD_P26 (Figure 3C). Notably, three genes outside of the deletions (*JAM2*, *ADAMTS1*, and *ETS2*) and within the Down syndrome critical region (DSCR) (Mowery et al., 2018) were downregulated in RD_P26 NESCs when compared to euploid NESCs.

We next sought to investigate the relative expression levels of genes from the ring chromosome and the non-deleted homologue in RD_P26 NESCs. To this end, we used the RNA-Seq data to identify high quality heterozygous SNVs in transcripts from chromosome 21. We identified 107 such SNVs on the ring chromosome outside the SVs. All heterozygous SNVs had an expected fraction at around 0.5 suggesting similar expression levels of genes from the ring chromosome and the non-deleted homologue (Supplementary Figure S3).

### Panther Pathway Analysis for Genome-wide Expression Pattern

We then analyzed the genome-wide DEGs (n = 1951) for enrichment in GO biological process using Panther pathway analysis (Mi et al., 2019). After filtering against an FDR >0.001, we derived a list of 296 pathways with a fold enrichment ranging from 0.06 to 4.8, median = 1.9 (Supplementary Table S3). Upon the highest ranked groups, there were “autonomic nervous system development”, GO:0048483, 4.4-fold; “positive regulation of axonogenesis”, GO:0050772, 3.7-fold; “stem cell proliferation”, GO:0072089, 3.7-fold; and “nerve development”, GO:0021675, 3.5-fold (Figure 3D). Pathways analyses, conducted on DEGs from either chromosome 21 or deleted regions alone, did not result in any significant finding.

### Gene Expression of Ring Chromosome Mirrors Trisomy 21

**FIGURE 4:**
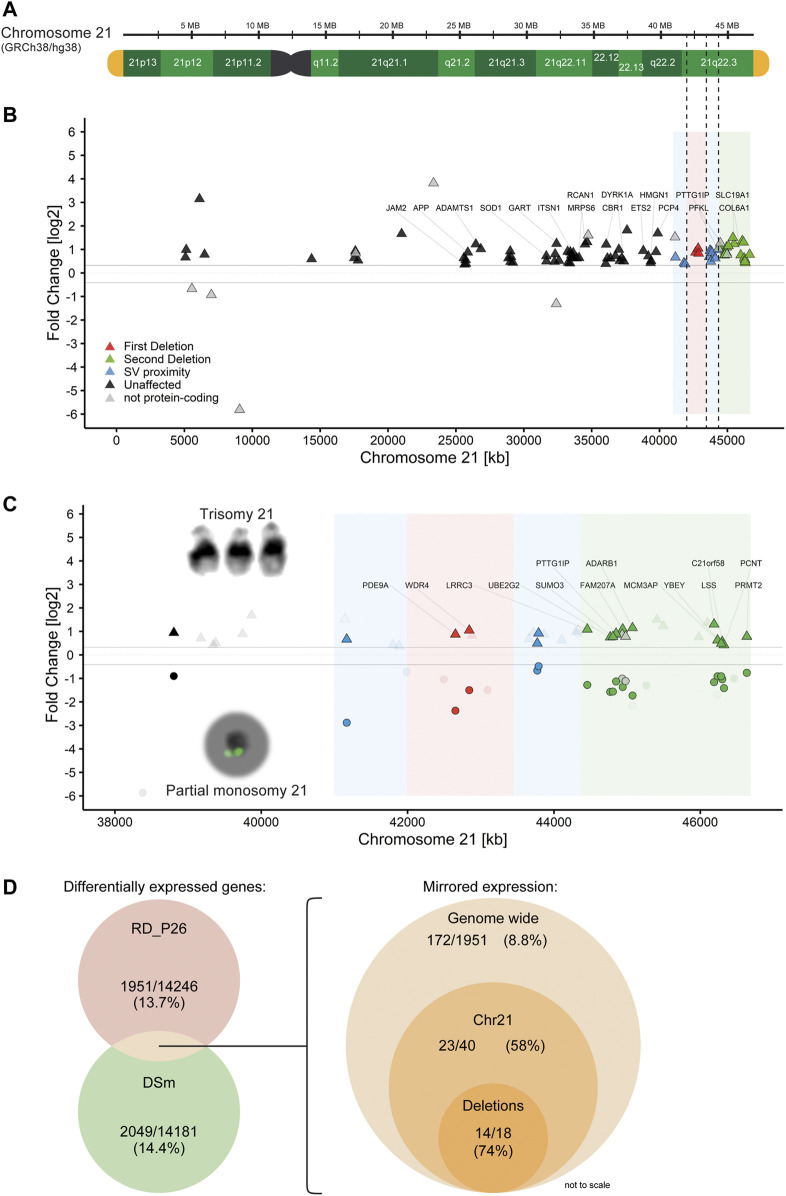
Partial monosomy 21 acts as transcriptomic mirror to trisomy 21 **(A)** Reference genome GRCh38/hg38 shown with G-bands (green), telomeres (yellow) and deletions from RD_P26 (dashed line) **(B)** Gene expression of the trisomy 21 sample DSm on chromosome 21. Shown genes are significantly and differentially (±25%) expressed. Genes related to Down Syndrome (Supplementary Table S7) are labelled. Deletions (red, green) and 1 Mb vicinity (blue) of RD_P26 are shown as color sections **(C)** Gene expression of significantly and differentially expressed genes grouped for DSm (triangles) and RD_P26 (circles). Genes were marked for protein-coding + mirrored (satiated color), non-coding genes (gray) and not-shared among the two samples (transparent). Only genes within the two deletions are labelled with the respective gene names. Note that all mirrored genes of DSm and RD_P26 are all upregulated and downregulated, respectively, and therefore locate above and below the limit for differential expression (±25%) **(D)** Venn diagrams providing numbers for significantly and differentially expressed (left) genes in RD_P26 (red) and DSm (green) and mirrored expression (right) for shared genes, displayed for genome-wide, chromosome 21 and the two deletions. The size of circles is not to scale.

Trisomy for chromosome 21 is associated with global transcriptomic changes. Since trisomy 21 represents the genetic contrary to monosomy 21, we therefore sought to compare the global transcriptional changes in RD_P26 NESCs with that in the trisomic DS1 and DS2 NESCs. To this end, we first generated RNA-Seq data from the NESC lines DS1 and DS2 (Sobol et al., 2019). The data was merged (DSm) to eliminate variations from each of the biological replicate, fed into DeSeq2 and compared to the RNA-Seq data generated from euploid NESCs using the same pipeline as for the analysis of RD_P26. The analysis revealed 14.4% DEGs (2049 of 14181) (Supplementary Figure S2B, raw data in Supplementary Table S4). The genome-wide expression data for individual trisomy 21 NESC lines are given in Supplementary Tables S5,6. Among all DEGs on chromosome 21, 84 out of 141 (59%) showed a relative upregulation in DSm (Figures 4A,B). Furthermore, 27 Down Syndrome risk genes (Brodsky et al., 1997; Peled-Kamar et al., 1998; Yamakawa et al., 1998; Chrast et al., 2000; Barlow et al., 2001; Levanon et al., 2001; Bell et al., 2003; Wolvetang et al., 2003; Martínez de Lagrán et al., 2004; Rainis et al., 2005; Arron et al., 2006; Belichenko et al., 2007; Sultan et al., 2007; Mullighan et al., 2009; Chakrabarti et al., 2010; Perluigi and Butterfield, 2011; Wu et al., 2014; Kazemi et al., 2016; Raveau et al., 2017; Mowery et al., 2018; Valbuena et al., 2019) reported in the literature both outside and inside of the DSCR (Supplementary Table S7) were all upregulated in DSm as well as of 19 out of the 31 expressed genes (61%) located within the deleted regions in RD_P26 (Supplementary Table S8). We observed differential gene expression in both RD_P26 and DSm for *JAM2*, *ADAMTS1*, *SOD1*, *ITSN1*, *ETS2* and *PTTG1IP*. Of those, only *PTTG1IP* is deleted in RD_P26.

Next, we compared the transcriptional dysregulations in DSm NESCs with those in RD_P26 NESCs. We focused on genes with differential expression in both RD_P26 and DSm (Figure 4C, raw data in Supplementary Table S9). The analysis revealed an overlap of 486 DEGs of which 172 showed an opposite expression pattern in RD_P26 and DSm relative to euploid NESCs. The number represents 8.8% (172 of 1951) of all differentially expressed genes in RD_P26. A similar and opposite expression pattern was observed for 58% of the DEGs on chromosome 21 (23 of 40) and of 74% of genes (14 of 18) deleted on the ring chromosome (Figure 4D; Table 1). When excluding the 18 genes within the deletions, 7.8% of all genes (154 of 1951) showed opposite expression pattern in DSm vs RD_P26 relative euploid NESCs (Permutation tests, n = 1,000, mean mirrored fraction = 0.069, std. error <0.0001, *p* = 0.0011). These data suggest a global effect on transcription caused by the deleted regions on chromosome 21 that to a large extent opposes to the transcriptional dysregulation caused by trisomy for chromosome 21.

**TABLE 1:** Expression of mirrored genes on chromosome 21 (RD_P26 compared to DSm). Genes were filtered for significantly differential expression and marked if related to Down Syndrome (bold, unfiltered list in Supplementary Table S7) and hemizygous in RD_P26 (underlined, unfiltered list in Supplementary Table S9).

Gene Name	Start coord. Chr21 (hg38)	RD_P26			DSm		
		Fold Change (log2)	Fold Change (Linear)	*p*-value (Bonferroni corrected)	Fold Change (log2)	Fold Change (Linear)	*p*-value (Bonferroni corrected)
MRPL39	25,585,656	−0.59	0.67	<0.001	0.63	1.55	<0.001
**JAM2**	25,639,258	−0.42	0.75	0.011	0.36	1.29	0.017
CYYR1	26,466,209	−0.80	0.57	0.043	0.43	1.34	<0.001
**ADAMTS1**	26,835,755	−1.02	0.49	0.002	1.02	2.03	<0.001
SCAF4	31,671,000	−0.47	0.72	<0.001	0.50	1.41	<0.001
**ETS2**	38,805,183	−0.90	0.54	0.003	0.94	1.92	<0.001
BACE2	41,167,801	−2.88	0.14	<0.001	0.78	1.72	<0.001
PDE9A	42,653,621	−2.38	0.19	<0.001	0.87	1.83	<0.001
WDR4	42,843,094	−1.50	0.35	<0.001	1.04	2.06	<0.001
CSTB	43,772,511	−0.67	0.63	<0.001	0.49	1.41	0.006
RRP1	43,789,513	−0.48	0.72	0.007	0.92	1.89	<0.001
LRRC3	44,455,510	−1.28	0.41	<0.001	1.09	2.12	<0.001
UBE2G2	44,768,580	−1.58	0.34	<0.001	1.09	2.13	<0.001
SUMO3	44,805,617	−1.56	0.34	<0.001	1.30	2.46	<0.001
**PTTG1IP**	44,849,585	−1.13	0.46	<0.001	0.63	1.54	<0.001
FAM207A	44,940,012	−1.37	0.39	0.001	0.56	1.47	0.013
ADARB1	45,073,853	−1.73	0.30	<0.001	1.22	2.33	<0.001
LSS	46,188,141	−1.16	0.45	<0.001	0.66	1.58	0.024
MCM3AP	46,235,133	−0.91	0.53	<0.001	0.48	1.40	0.044
YBEY	46,286,342	−0.91	0.53	<0.001	0.91	1.88	<0.001
C21orf58	46,300,181	−1.04	0.49	<0.001	0.75	1.69	<0.001
PCNT	46,324,141	−1.41	0.38	<0.001	0.77	1.71	<0.001
PRMT2	46,635,595	−0.76	0.59	<0.001	1.15	2.22	<0.001

## Discussion

The ring chromosome 21 reported here (patient RD_P26) contains two deleted regions on chromosome 21qter resulting in the heterozygous loss of 60 protein-coding genes. Based on our established cellular model (Kvarnung et al., 2019; Sobol et al., 2019), we generated patient-specific neural stem cells to utilize whole transcriptome sequencing to delineate the impact on gene expression of partial monosomy 21 compared to trisomy 21. The patient had among other phenotypes a persistent impressive and expressive speech delay, which is why we used a neural cell model instead of only blood or fibroblasts. In this model, we show that both deleted and non-deleted genes have a lower expression. When focusing on the DEGs in NESCs with monosomy 21 and trisomy 21, 172 genes were identified presenting an opposed (“mirrored”) transcriptional pattern among a total set of 486 shared DEGs.

Reprogramming of somatic cells into iPSCs opens up new opportunities for understanding cellular disease mechanisms (Zakrzewski et al., 2019). While genomic changes detected in DNA are stable in different cell types, gene expression data are highly variable between tissues (Gonorazky et al., 2019) and to investigate the brain specific cells, a neural cell model such as NESCs, is needed.

Prior studies have shown that trisomy 21 impacts the gene expression levels on both chromosome 21 and genome-wide (Lockstone et al., 2007; Sobol et al., 2019). The transcriptome data generated from NESCs retaining the ring chromosome 21 provides a unique opportunity to understand how monosomy for one third of genes on chromosome 21 affects transcriptional changes during early neurogenesis.

We found that among 18 differentially expressed genes in the deleted regions, 14 have a “mirrored” expression profile in RD_P26 (downregulated) vs trisomic lines (upregulated). Two protein-coding genes, *COL6A2* and *NDUFV3*, are not differentially expressed despite being located in the deleted region. However, both genes are lower expressed compared to controls and it is not possible to conclude that the hemizygous expression is upregulated to compensate for the heterozygous loss. Of the 18 DEGs in the deleted regions, eight genes are reported with loss of function mutations without any phenotypic association (Supplementary Table S10) and homozygous deletions vary from no phenotype to lethal. Taken together, available data do not support that haploinsufficiency of any of the deleted genes has a major and direct effect on the phenotype of our case.

Our data revealed a genome-wide differential expression in 13.7% (1951 out of 14246) and 14.4% (2049 out of 14181) of genes in NESCs with ring chromosome 21 (RD_P26) and Trisomy 21 (DSm), respectively. An opposite gene expression pattern, with upregulation in DSm and downregulation in RD_P26 or vice versa, was observed for 172 genes (8.8%) when comparing the RD_P26 data to DSm. This finding highlights that “mirrored” genomic copy number variation, i.e. monosomy versus trisomy, have impacted the global gene expression. Our results may however pinpoint specific genes and pathways in the Down syndrome neuropathogenesis. However, it is important to consider that trisomy 21 affects all 237 genes on chromosome 21, and that only 25% (60 of 237) of those are deleted in RD_P26. Furthermore, only 25% (486 of 1951) of all differentially expressed genes in RD_P26 are also differentially expressed in DSm. It is therefore likely that the global transcriptomic changes detected in our model may interfere with critical developmental processes leading to the clinical features in our case.

One clear limitation of this study is that it examines only one monosomy NESC line. However, as seen by our permutation analysis, the number of mirrored genes in patient RD_P26 was significantly higher than compared to a random distribution of mirrored genes (7.8 vs. 6.9%, *p* = 0.0011). The same experimental design, to compare the expression profile of an individual with partial monosomy 21 to trisomy 21, could be scaled up. By performing similar studies on other individuals with deletions of various parts of chromosome 21 a complete map of transcriptional effects of monosomy 21 might be constructed. Such an approach will also show us which chromosome 21 genes are mirrored compared to trisomy 21 as well as global effects of those genes.

In general, the use of NESCs as neural cell model is known to be adequate featuring the reproducibility of results despite the variability detected by RNA-Seq (Kvarnung et al., 2019; Sobol et al., 2019). As an example, the overlap of differentially expressed genes with our analysis of transcriptomes from trisomic NESCs is only 8.6% (239 of 2049) (Sobol et al., 2019). This rather low overlap is explained by the low number of individuals in our study as well as by the differences in specific time points of extraction leading to higher biological variability. The experimental setup involving control cells as well as the data cleaning also plays an important role. As a consequence, the overlap of DEGs from different studies is not smaller than expected.

## Conclusion

We present herein a case with neurodevelopmental delay, feeding and growth problems, speech delay and hemifacial microsomia associated with chromosome 21qter deletions caused by a ring chromosome 21. Early neural development was modeled in subject-derived NESCs, and RNA-Seq revealed global transcriptional changes associated with the deletions. No single deleted gene was identified as a strong candidate for any of the clinical features in our case and it is likely that the neurodevelopmental phenotype is caused by a combined effect of the 18 downregulated and deleted genes, together with the genome-wide expression changes. Furthermore, using RNA-Seq on patient-derived NESCs, we were able to pinpoint genes with a genome-wide effect on gene expression that might warrant further study. Follow up studies with more patients and with different overlapping deletions or duplications on chromosome 21 may allow for a dissection of the gene regulatory network involving chromosome 21.

## Data Availability

The datasets presented in this study can be found in online repositories. The names of the repository/repositories and accession number(s) can be found below: www.ncbi.nlm.nih.gov/, GSE190053.
