# Supplementary figures and images for: Partial Monosomy 21 Mirrors Gene Expression of Trisomy 21 in a Patient-Derived Neuroepithelial Stem Cell Model
Source: Front Genet. 2022 Feb 4;12:803683. doi: 10.3389/fgene.2021.803683 (PMC8854775; doi:10.3389/fgene.2021.803683)

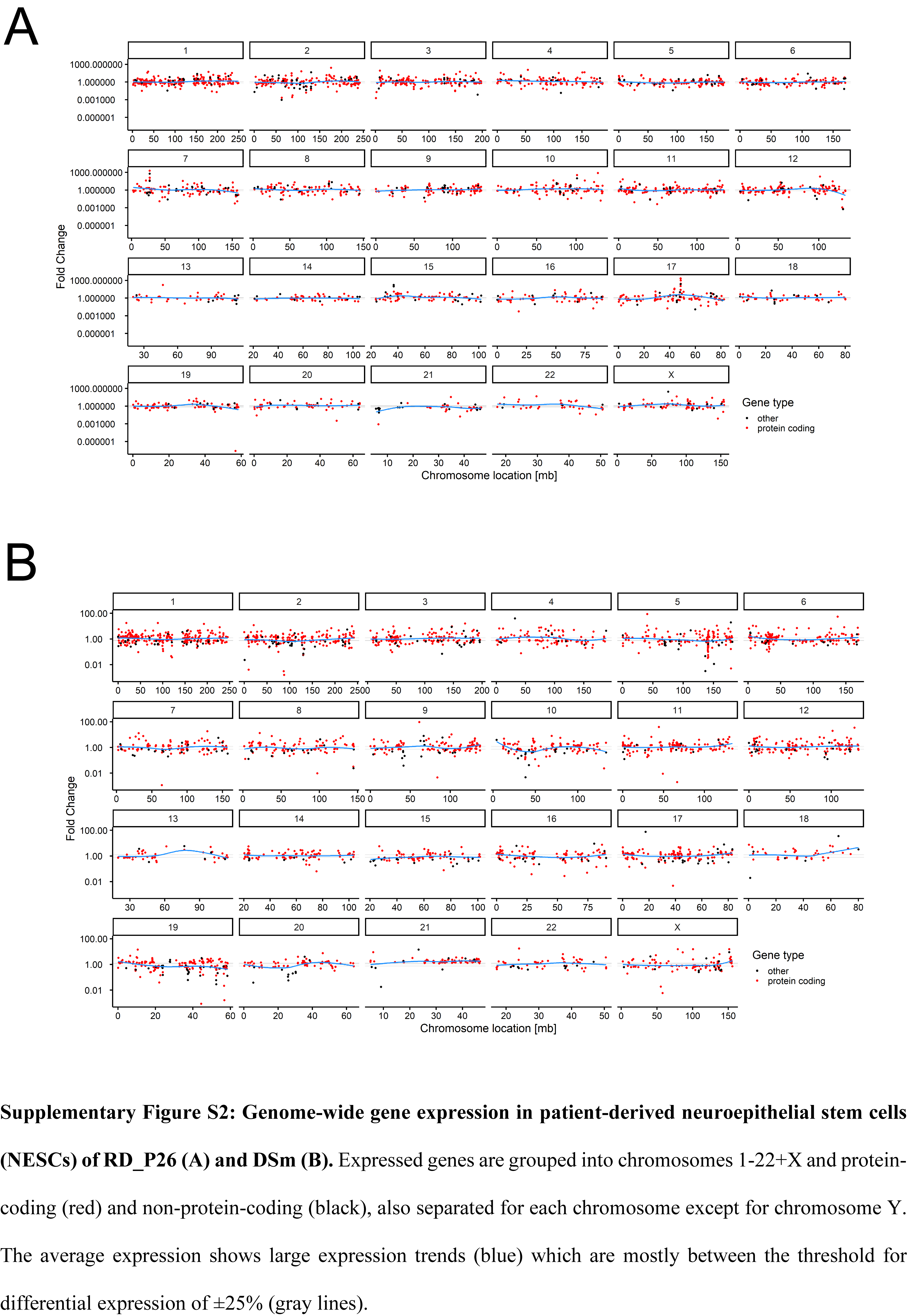

Supplement: Supplementary file 1 [file Image2.png]

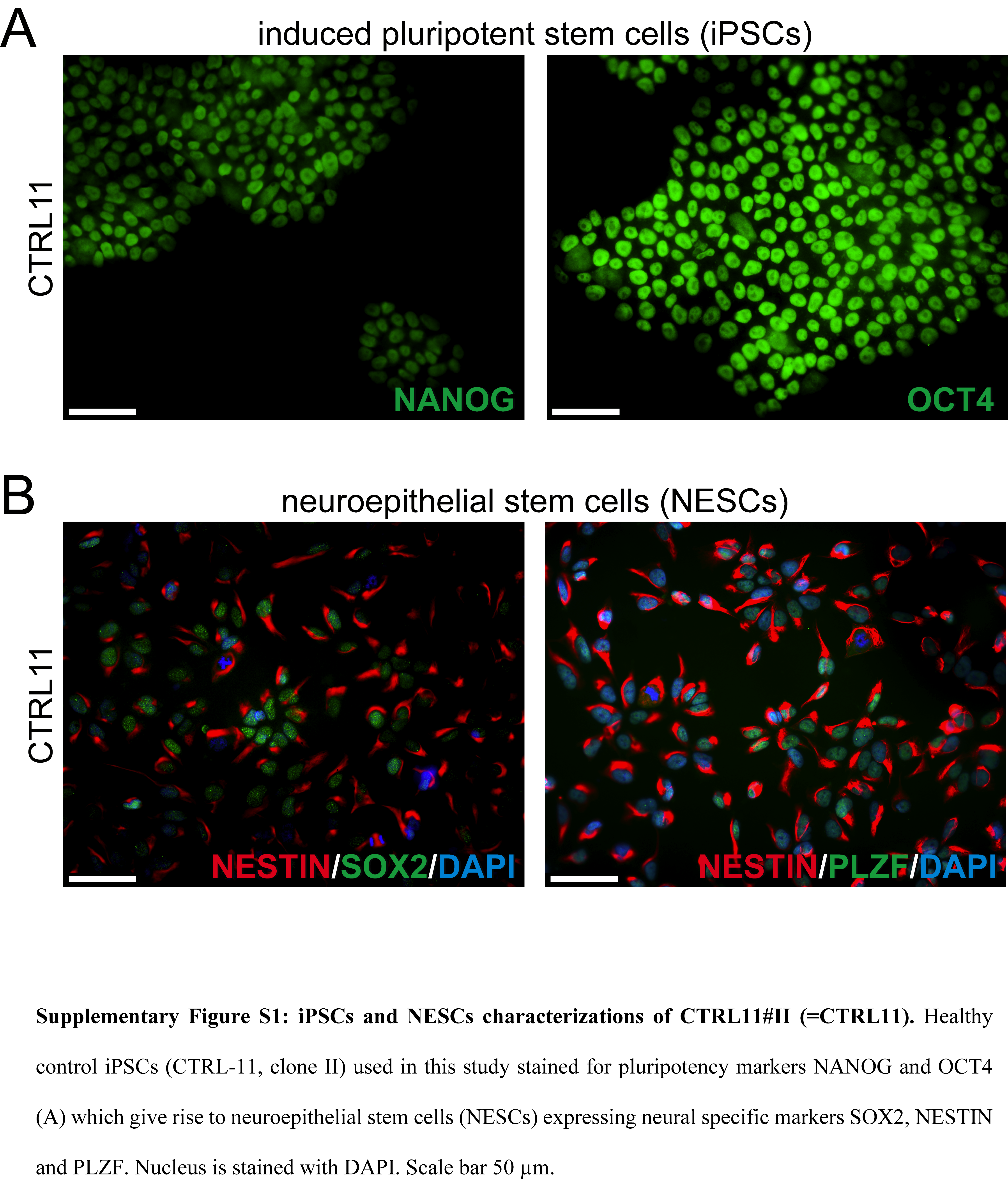

Supplement: Supplementary file 2 [file Image1.png]

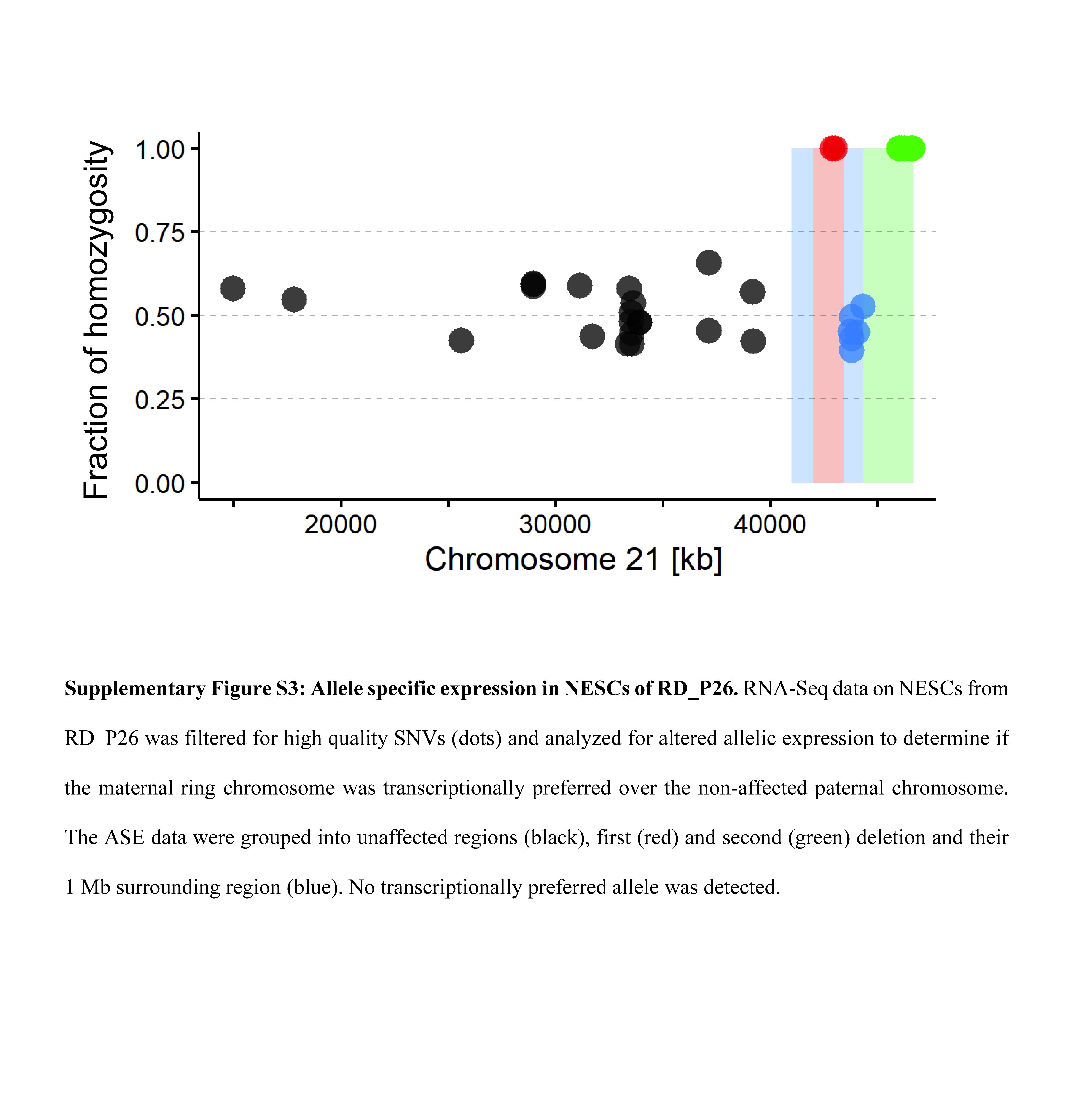

Supplement: Supplementary file 3 [file Image3.png]
